# Unemployment Insurance for Nurses

**Published:** 1920-02-21

**Authors:** 


### THE MATRONS' AND SISTERS' DEPARTMENT.

### UNEMPLOYMENT INSURANCE FOR NURSES.

It is a matter of some moment for nurses that they shall not be left outside the provisions of Bills aimed at diminishing the risks and impediments which lie in the path of wage earners. Among the measures devised for ameliorating the lot of those on whom the burden of the world's work rests, none is more necessary than protection from the evils of intermittent employment.

Whenever want overtakes nurses after middle age, experience shows that the breakdown of their independence has gradually been accomplished by slow degrees as a consequence of long periods of fluctuating employment, during which their previous savings were consumed to meet the long gaps between cases. There is a very large floating population of nurses who get cases from institutes or agents or private doctors. When the season is what Mr. Woodhouse called "sickly" these nurses are all wanted and many more could be absorbed by the public. But happily for the public these "sickly seasons" are infrequent. In normal times the demand for nurses falls far below this high-water mark. And every year there is a month or two during which it falls to low-water mark, when there is hardly any business stirring at all. These seasonal fluctuations are familiar to all acquainted with nursing work. They form the reason why nurses of a thrifty turn of mind are wont to prefer even a very moderate salary under an inclusive living-in system rather than expose themselves to the risk of having to support themselves for indefinite periods without pay.

It ought to be realised by all classes of nurses that these are conditions which point to the need for unemployment insurance. In the public interest it is necessary to have a far larger body of private nurses in readiness than can normally find continuous employment. By a natural process of selection it is the younger and more recently trained that will be preferred when a choice has to be made from among a group of competing nurses all perhaps equally well fitted to undertake a case. The result is that the cry "too old at forty" is raised, a cry which is shown to be manifestly fictitious when a rush of work comes and patients consider themselves only too fortunate in securing one of the so-called "too old" nurses to attend to them.

Since it is for the public interest that the number of private nurses should be kept up to a figure beyond what is usually required, it is manifestly unfair that nurses should be allowed to suffer when they are exposed for this cause to unemployment. It is quite certain that under a thoroughly well-organised system of nursing this difficult matter could be wisely regulated. The nurse who joins a living-in institute or co-operation does by that very act insure herself against unemployment, but often by a far greater sacrifice of income than is just. There are at present no means whereby the women who vastly prefer to choose their own place of residence can be safeguarded effectually from the normal fluctuations of their profession. We are disposed to believe that their inclusion, with special rules to suit their special calling, in the Bill which is being brought before Parliament in the present session might have the desired effect. But in any case, the matter is one which requires more attention than has hitherto been bestowed upon it.

Nurses are not as a rule keenly alive to their own worldly interests. It is much to be feared that young nurses when confronted with the duty of paying in to an insurance fund intended to secure them from the future contingency of meeting with long periods of unemployment, may be disposed to shirk the issue. Nothing seems so unlikely to a nurse in the first rush of remunerative work as that her day will soon be over and others be found treading on her heels. If she contemplate the future at all she is apt to regard it with rose-coloured spectacles. A little gentle compulsion in the art of providing for the lean years is needed if the nursing profession is to be rescued from the burden of supporting such of its members as have neglected to make timely provision for the lessening of their earning capacity after the age of forty-five.

There can be no question that nursing is one of those special occupations which need very special organisation. Measures which do very well for manual workers may not entirely meet the case for nurses. For that reason it is incumbent on the leaders of the profession to look into this matter of unemployment insurance very carefully while there is still time. If nurses are not included in the operations of the Unemployment Bill none but persuasive and non-compulsory methods can be applied to rescue them from impecuniosity. Now it has been pretty conclusively proved that persuasive methods applied to a great body of independent workers are of very little good.

